# The Role of Peroxisome Proliferator-Activated Receptor *β*/*δ* on the Inflammatory Basis of Metabolic Disease

**DOI:** 10.1155/2010/368467

**Published:** 2010-07-27

**Authors:** Teresa Coll, Emma Barroso, David Álvarez-Guardia, Lucía Serrano, Laia Salvadó, Manuel Merlos, Xavier Palomer, Manuel Vázquez-Carrera

**Affiliations:** Department of Pharmacology and Therapeutic Chemistry, CIBER de Diabetes y Enfermedades Metabólicas Asociadas (CIBERDEM)-Instituto de Salud Carlos III and Institut de Biomedicina de la Universitat de Barcelona (IBUB), Faculty of Pharmacy, University of Barcelona, Diagonal 643, 08028 Barcelona, Spain

## Abstract

The pathophysiology underlying several metabolic diseases, such as obesity, type 2 diabetes mellitus, and atherosclerosis, involves a state of chronic low-level inflammation. Evidence is now emerging that the nuclear receptor Peroxisome Proliferator-Activated Receptor (PPAR)*β*/*δ* ameliorates these pathologies partly through its anti-inflammatory effects. PPAR*β*/*δ* activation prevents the production of inflammatory cytokines by adipocytes, and it is involved in the acquisition of the anti-inflammatory phenotype of macrophages infiltrated in adipose tissue. Furthermore, PPAR*β*/*δ* ligands prevent fatty acid-induced inflammation in skeletal muscle cells, avoid the development of cardiac hypertrophy, and suppress macrophage-derived inflammation in atherosclerosis. These data are promising and suggest that PPAR*β*/*δ* ligands may become a therapeutic option for preventing the inflammatory basis of metabolic diseases.

## 1. Introduction

Over the last decade, an abundance of evidence has shown a close link between a state of chronic low-level inflammation and metabolic dysfunction. In fact, excessive nutrition consumption or storage has the capacity to activate both inflammatory and metabolic signaling networks since they are linked and interdependent [1].

Peroxisome Proliferator-Activated Receptors (PPARs), which are members of the nuclear receptor family, have emerged as important regulators of metabolic and inflammatory signaling, particularly in the context of metabolic disease [2–4]. The ability of these receptors to connect metabolism and inflammation makes them interesting targets for the treatment of metabolic diseases, such as atherosclerosis and diabetes, through modulation of the inflammatory process. Here, we will focus on recent advances in our understanding of the role of one of these PPAR members, the PPAR*β*/*δ*, as an integrator of metabolic and inflammatory signaling networks.

## 2. Peroxisome Proliferator-Activated Receptors (PPARs)

**Figure 1:**
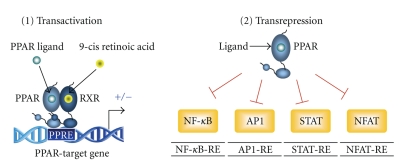
Molecular mechanisms of Peroxisome Proliferator-Activated Receptors (PPARs). PPARs are ligand-activated transcription factors that regulate gene expression through two mechanisms: transactivation and transrepression. In transactivation, PPAR-RXR heterodimers bind to DNA-specific sequences called peroxisome proliferator-response elements (PPREs), which are located in the promoter regions of genes involved in glucose and fatty acid metabolism. PPARs may also regulate gene expression through a DNA-independent mechanism called transrepression. Through this mechanism, PPARs inhibit the activity of several transcription factors such as Nuclear Factor-*κ*B, which leads to anti-inflammatory effects. STAT denotes signal transducers and activators of transcription, IS-GFRE is the interferon-stimulated gene factor responsive element, and TRE is the TPA responsive element, where TPA is a phorbol ester.

PPARs are members of the nuclear receptor superfamily of ligand-activated transcription factors that regulate the expression of genes involved in fatty acid uptake and oxidation, lipid metabolism, and inflammation [2]. To be transcriptionally active, PPARs need to heterodimerize with the 9-*cis *retinoic acid receptor (RXR) (NR2B) (Figure 1). PPAR-RXR heterodimers bind to DNA-specific sequences called peroxisome proliferator-response elements (PPREs), which consist of an imperfect direct repeat of the consensus binding site for nuclear hormone receptors (AGGTCA), separated by one nucleotide (Direct Repeat 1, DR-1). These sequences have been characterized within the promoter regions of PPAR target genes. The binding occurs in such a way that PPAR is always oriented to the DNA's 5′-end, while RXR is oriented to the 3′-end. In the absence of a ligand, high-affinity complexes are formed between PPAR-RXR heterodimers and nuclear receptor corepressor proteins, which block transcriptional activation by sequestering the heterodimer from the promoter. 

In a specific cellular context, the activity of PPARs that regulate the transcription of their target genes depends on many factors (relative expression of the PPARs, the promoter context of the target gene, the presence of coactivator, and corepressor proteins, etc.). Thus, the transcriptional activity of PPARs is modulated by coactivators and corepressors [5]. One of the best described PPAR coactivators is PPAR*γ* coactivator 1*α* (PGC-1*α*). Silencing mediator for retinoic and thyroid hormone receptor (SMRT) and the nuclear receptor corepressor are corepressors that interact with the PPARs in the absence of ligands [6]. Receptor-interacting protein 140 (RIP140), an important metabolic regulator, is another ligand-dependent corepressor which interacts with PPARs. Binding of the ligand to PPAR induces a conformational change resulting in dissociation of corepressor proteins, so that the PPAR-RXR heterodimer can then bind to PPREs. Moreover, once activated by the ligand, the heterodimer recruits coactivator proteins that promote the initiation of transcription [4]. As a result of these changes in transcriptional activity, binding of ligands to the receptor leads to changes in the expression level of mRNAs encoded by PPAR target genes. 

Finally, PPAR activity is also regulated at the posttranscriptional level by phosphorylation, ubiquitinylation, and sumoylation (for a detailed review, see [5]).

However, the regulation of gene transcription by PPARs extends beyond their ability to transactivate specific target genes. PPARs also regulate gene expression independently of binding to PPREs. They cross-talk with other types of transcription factors and influence their function without binding to DNA, through a mechanism termed receptor-dependent* trans-*repression [7]. Most of the anti-inflammatory effects of PPARs can probably be explained in this way [8, 9]. Through this DNA-binding independent mechanism, PPARs suppress the activities of several transcription factors, including nuclear factor *κ*B (NF-*κ*B), activator protein 1 (AP-1), signal transducers and activators of transcription (STATs), and the nuclear factor of activated T cells (NFAT). There are three main transrepression mechanisms by which ligand-activated PPAR-RXR complexes negatively regulate the activities of other transcription factors. First, transrepression may result from competition for limited amounts of shared coactivators. Under conditions in which the levels of specific coactivators are rate-limiting, activation of PPAR may suppress the activity of other transcription factors that use the same coactivators [10, 11]. In the second mechanism, activated PPAR-RXR heterodimers are believed to act through physical interaction with other transcription factors (e.g., AP-1, NF-*κ*B, NFAT, or STATs). This association prevents the transcription factor from binding to its response element and thereby inhibits its ability to induce gene transcription [12]. The third transrepression mechanism relies on the ability of activated PPAR-RXR heterodimers to inhibit the phosphorylation and activation of certain members of the mitogen-activated protein kinase (MAPK) cascade [13], which prevents activation of downstream transcription factors.

The PPAR family consists of three members, PPAR*α* (NR1C1 according to the unified nomenclature system for the nuclear receptor superfamily), PPAR*β* */* *δ* (NR1C2) and PPAR*γ* (NR1C3) [14]. PPAR*α* was the first PPAR to be identified and is the molecular target of the fibrate hypolipidemic class of drugs. This PPAR isotype is expressed primarily in tissues that have a high level of fatty acid catabolism, such as liver, brown fat, kidney, heart, and skeletal muscle [15]. PPAR*γ* has a restricted pattern of expression, mainly in white and brown adipose tissue and colon, and is also expressed in macrophages. Other tissues, such as skeletal muscle and heart, contain only limited amounts. PPAR*β* */* *δ* is ubiquitously expressed and for this reason it was initially considered a “housekeeping gene” [16]. However, studies with knockout mice [17–19] and the development of specific and high-affinity ligands for this receptor have shown that PPAR*β* */* *δ* is a potential molecular target to prevent or treat several metabolic disorders. 

Natural ligands for the PPARs are believed to include native and modified polyunsaturated fatty acids and eicosanoids. Since the discovery of PPARs, a number of synthetic ligands for these receptors have been identified [20]. Thus, fibrates activate PPAR*α*, whereas the antidiabetic drugs thiazolidinediones activate the *γ* isotype [21, 22].

## 3. PPAR**β**/**δ**-Specific Features and Ligands

The crystal structure of the ligand-binding domain of the PPAR*β* */* *δ* isotype, which was first cloned in *Xenopus laevis *[23], revealed an exceptionally large pocket of approximately 1300 Å^3^. This pocket is similar to that of PPAR*γ*, but much larger than the pockets of other nuclear receptors [24, 25]. This may partially explain the great variety of natural and synthetic ligands that bind to and activate this nuclear receptor. Saturated (14 to 18 carbons) and polyunsaturated (20 carbons in length) fatty acids have affinities for PPAR*β* */* *δ* in the low micromolar range [25–28]. In addition, all-*trans*-retinoic acid (vitamin A) [29] and fatty acids derived from very low density lipoprotein (VLDL) [30] can activate PPAR*β* */* *δ*. Finally, the number of experimental studies on the role of PPAR*β* */* *δ* in cellular processes increased greatly as a result of the availability of several synthetic ligands (including GW501516, GW0742, and L-165041) that activate PPAR*β* */* *δ* at low concentrations both *in vivo* and *in vitro* and have high selectivity over other PPAR isotypes [31]. The EC_50_ for these compounds, which were assessed with recombinant human PPAR*β* */* *δ*, were 1.0 nM for GW0742, 1.1 nM for GW501516, and 50 nM for L-165041 [31, 32]. Recently, the first PPAR*β*/*δ* synthetic antagonist (GSK0660) has been identified [33].

## 4. PPAR**β**/**δ**, Inflammation, and Adipose Tissue

The expansion of adipose tissue, mainly in the form of visceral obesity, may contribute to enhanced inflammation in this tissue through several processes. First, macrophages can infiltrate in adipose tissue, which contributes to the overproduction of inflammatory cytokines, such as tumor necrosis factor *α* (TNF-*α*) and interleukin 6 (IL-6) [34–36]. Indeed, the infiltration of macrophages into adipose tissue correlates with the degree of insulin resistance [34]. Second, as visceral fat (which is very sensitive to lipolytic stimuli) increases, so does the rate of lipolysis. This leads to increased free fatty acid (FFA) mobilization and elevated levels of circulating FFA. Several studies have consistently demonstrated that elevations of plasma FFA produce insulin resistance in diabetic patients and in nondiabetic subjects [37–39]. Saturated FFA are potent activators of the Toll-like receptor-4 (TLR4) [40] and recent evidence suggests that inflammatory processes induced by obesity and a high-fat diet cause systemic insulin resistance via a mechanism involving this receptor [41]. TLR-4 is expressed in virtually all human cells and binds a wide spectrum of exogenous and endogenous ligands, including bacterial lipopolysaccharide (LPS) [42]. In the presence of LPS, the TLR4 complex (including CD-14 and an accessory protein, MD-2) recruits the adaptor protein, myeloid differentiation factor-88 (MyD88), which in turn recruits interleukin-1 receptor-associated kinase (IRAK). This leads to the activation of the proinflammatory transcription factor NF-*κ*B [43] and the subsequent enhanced expression of several inflammatory mediators (including IL-6 and monocyte chemoattractant protein-1 [MCP-1]). These observations indicate that saturated FFA derived from adipocytes and from high-fat diets activate TLR and the inflammatory pathway in adipocytes and macrophages, which contribute to the synthesis and production of cytokines such as TNF-*α* [44]. In addition, high-fat diets raise plasma LPS to a concentration that is high enough to increase body weight, fasting glycemia, and inflammation [45]. Furthermore, LPS receptor-deleted mice (CD14 mutants) are hypersensitive to insulin, and the development of insulin resistance, obesity, and diabetes in this animal model is delayed in response to a high-fat diet [45]. Experiments performed in our laboratory have demonstrated that the PPAR*β*/*δ* agonist GW501516 inhibits LPS-induced cytokine expression and secretion by preventing NF-*κ*B activation in adipocytes [46]. Of note, NF-*κ*B activation by LPS requires mitogen-activated protein kinase (MAPK)-extracellular signal-related kinase (ERK)1/2 (MEK1/2) activation, since inhibition of this pathway reduces LPS-induced cytokine production in adipocytes [47]. In agreement with this role of ERK1/2 in inflammation in adipocytes, the expression of proinflammatory cytokines in these cells drops when they are exposed to LPS in the presence of the MAPK pathway inhibitor U0126. Interestingly, in white adipose tissue from PPAR*β*/*δ*-null mice we observed increased ERK1/2 phosphorylation and NF-*κ*B activity and higher expression of IL-6 compared with wild-type mice [46]. Moreover, in the white adipose tissue of a genetic model of obesity and diabetes, the Zucker diabetic fatty (ZDF) rat, the reduction in the expression of PPAR*β*/*δ* correlated with an increase in ERK1/2 phosphorylation and NF-*κ*B activity. These findings suggest that PPAR*β*/*δ* activation prevents LPS-induced NF-*κ*B activation via ERK1/2, thereby reducing the production of proinflammatory cytokines involved in the development of insulin resistance. 

In addition, PPAR*β*/*δ* is involved in the phenotypic switch of adipose tissue-resident macrophages that modulates insulin sensitivity [48]. Thus, it has been suggested that macrophages infiltrated in adipose tissue from lean animals show an alternatively activated M2 phenotype [49] that is induced by Th2 cytokines, such as IL-4 and IL-13. These macrophages produce IL-10, a cytokine that inhibits inflammation [50]. In contrast, high-fat diets lead to infiltration of macrophages that show markers of classic activation by Th1 cytokines, such as TNF-*α* and IL-1*β*. These M1 phenotypic macrophages produce proinflammatory cytokines that lead to metabolic disturbances. In infiltrated macrophages in adipose tissue and liver, the signaling of Th2 cytokines is transduced by PPAR*β*/*δ* through a signal transducer and activator of transcription 6 (STAT6) binding site on its promoter, which induces alternative activation. The subsequent switch to the M2 phenotype prevents the inflammation caused by inflammatory mediators, such as FFA, in adipose tissue and liver. In agreement with this model, myeloid-specific PPAR*β*/*δ* ^−/−^ mice show adipocyte disfunction, insulin resistance, and hepatosteatosis [48].

## 5. PPAR**β**/**δ**, Inflammation, and Insulin Resistance in Skeletal Muscle Cells

FFAs may cause insulin resistance in skeletal muscle through several mechanisms, including effects on metabolism [51, 52], signaling [53, 54], and mitochondrial function [55, 56]. In addition, FFAs activate proinflammatory pathways, linking the development of this pathology to a chronic low-grade systemic inflammatory response [57]. In addition to FFA-induced inflammation through TLR, an additional pathway leads to FFA-mediated inflammation. This pathway involves intracellular accumulation of fatty acid derivatives. Once fatty acids are taken up by skeletal muscle cells they are either stored as fatty acid derivatives or undergo *β*-oxidation in the mitochondria. In the presence of high plasma FFA, fatty acid flux in skeletal muscle cells exceeds its oxidation, which leads to the accumulation of fatty acid derivatives, such as diacylglycerol (DAG), which can then activate a number of different serine kinases that negatively regulate insulin action. Thus, DAG is a potent allosteric activator of protein kinase C*θ* (PKC*θ*), which is the most abundant PKC isoform in skeletal muscle [58–60]. This PKC isoform inhibits the action of insulin by phosphorylating certain serine residues on insulin receptor substrate 1 (IRS1), including Ser^307^ in the rodent IRS-1 protein (reviewed in [61]). This phosphorylation impairs insulin-receptor signaling through several distinct mechanisms [62]. PKC*θ* also impairs insulin sensitivity by activating another serine kinase, I*κ*B kinase *β* (IKK*β*) [63]. In addition to phosphorylating IRS-1 in Ser^307^, IKK*β* phosphorylates I*κ*B. Thus, it activates the proinflammatory transcription factor NF-*κ*B, which has been linked to fatty acid-induced impairment of insulin action in skeletal muscle in rodents [64, 65]. Once activated, NF-*κ*B regulates the expression of multiple inflammatory mediators, including IL-6. This cytokine correlates strongly with insulin resistance and type 2 diabetes [66–68] and its plasma levels are 2-3 times higher in patients with obesity and type 2 diabetes than in lean control subjects [67].

**Figure 2:**
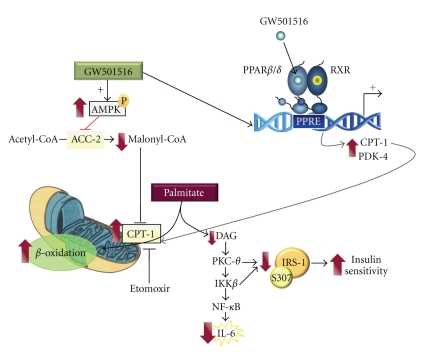
Potential mechanism of action involved in the reduction of insulin resistance and inflammation in skeletal muscle cells following PPAR*β*/*δ* activation by GW501516. ACC-2, acetyl-CoA carboxylase 2; AMPK, AMP-activated protein kinase; CPT-1, carnitine palmitoyltransferase-1; DAG, diacylglycerol; IKK*β*, I*κ*B kinase *β*; IL-6, interleukin 6; IRS-1, Insulin Receptor Substrate 1; NF-*κ*B, Nuclear Factor-*κ*B; PDK-4, pyruvate dehydrogenase kinase 4; PKC*θ*, protein kinase C*θ*.

Accumulation of fatty acid derivatives can be attenuated by mitochondrial *β*-oxidation. The rate-limiting step for *β*-oxidation of long-chain fatty acids is their transport into mitochondria via carnitine palmitoyltransferase-1 (CPT-1). The activity of this enzyme is inhibited by malonyl-CoA, the product of acetyl-CoA carboxylase, which, in turn, is inhibited by the AMP-activated protein kinase (AMPK). This kinase is a metabolic sensor that detects low ATP levels and increases oxidative metabolism [69], by reducing the levels of malonyl-CoA. Interestingly, activation of fatty acid oxidation by overexpressing CPT-1 in cultured skeletal muscle cells [70] and in mouse skeletal muscle [71] improves lipid-induced insulin resistance. Hence, this approach may provide a valid therapeutic strategy to prevent this pathology. Activation of PPAR*β*/*δ* by its ligands (including GW501516) enhances fatty acid catabolism in adipose tissue and skeletal muscle, thereby delaying weight gain (for a review, see [72]). This increase in fatty acid oxidation in human skeletal muscle cells following PPAR*β*/*δ* activation by GW501516 is dependent on both PPAR*β*/*δ* and AMPK [73]. AMPK is activated by GW501516 by modulating the ATP : AMP ratio [73]. Despite these data, little information was available on whether the increase in fatty acid oxidation attained after PPAR*β*/*δ* activation prevented fatty acid-induced inflammation and insulin resistance in skeletal muscle cells. However, we have recently reported that the PPAR*β*/*δ* ligand GW501516 prevented palmitate-induced inflammation and insulin resistance in skeletal muscle cells [74]. Treatment with GW501516 enhanced the expression of two-well known PPAR*β*/*δ*-target genes involved in fatty acid oxidation, CPT-1 and pyruvate dehydrogenase kinase 4 (PDK-4), and increased the phosphorylation of AMPK (Figure 2). This prevented the reduction in fatty acid oxidation caused by palmitate exposure. In agreement with these changes, GW501516 treatment reversed the increase in DAG and PKC*θ* activation caused by palmitate. These effects were abolished in the presence of the CPT-1 inhibitor etomoxir, thereby implicating increased fatty acid oxidation in the changes. Consistent with these findings, PPAR*β* */* *δ* activation by GW501516 blocked palmitate-induced NF-*κ*B DNA-binding activity. Likewise, drug treatment inhibited the increase in IL-6 expression caused by palmitate in C2C12 myotubes and human skeletal muscle cells, as well as the protein secretion of this cytokine. Overall, these findings indicate that PPAR*β*/*δ* attenuates fatty acid-induced NF-*κ*B activation and the subsequent development of insulin resistance in skeletal muscle cells by reducing DAG accumulation. To our knowledge no studies have assessed whether this mechanism operates in humans. However, since GW501516 increases CPT-1 expression and palmitate oxidation in human skeletal muscle [75, 76], this possibility merits further exploration.

## 6. PPAR**β**/**δ**, Inflammation, and Heart Function

The constant pumping of the heart requires a high energy supply, which is mainly met by fatty acids and glucose. The oxidation of fatty acids and glucose covers 65% and 30% of the energy demand of the adult heart, respectively [77]. The heart, in contrast to other tissues such as the brain, adapts its metabolism to substrate availability. For example, an increase in glucose utilization and a decrease in fatty acid oxidation is observed during cardiac hypertrophy and congestive heart failure [78–80]. PPAR*β* */* *δ* *.* is involved in the control of fatty acid oxidation in heart, which is similar to its role in skeletal muscle. We have reported that the levels of both PPAR*α* and PPAR*β* */* *δ* are reduced in pressure-overload cardiac hypertrophy [81]. Therefore, the fall in the expression of both PPAR subtypes during the development of cardiac hypertrophy may be necessary to downregulate the expression of genes involved in fatty acid metabolism. Interestingly, the changes that cardiac hypertrophy causes in the expression of genes involved in fatty acid metabolism were not observed when NF-*κ*B activity was inhibited [82]. These data pointed to the involvement of NF-*κ*B in the changes. Therefore, we evaluated whether mechanisms such as protein-protein interaction between NF-*κ*B and PPAR contribute to the changes in the expression of genes involved in cardiac fatty acid metabolism, in addition to the reported reduction in the expression of PPARs during cardiac hypertrophy [83]. Using both *in vitro* and *in vivo* models of cardiac hypertrophy, we studied the contribution of NF-*κ*B activation to the downregulation of fatty acid oxidation during this process. Stimulation of rat neonatal cardiomyocytes with phenylephrine (PE), which leads to NF-*κ*B activation [84], caused cardiac hypertrophy that was accompanied by a fall in the expression of PDK-4 and palmitate oxidation. Furthermore, the reduction in the expression of PDK-4 and fatty acid oxidation observed in PE-stimulated rat neonatal cardiomyocytes was restored by NF-*κ*B inhibitors. These findings and additional studies [85] pointed to the involvement of NF-*κ*B in the downregulation of fatty acid oxidation during the development of cardiac hypertrophy. In agreement with this idea, a study demonstrated that cardiomyocyte-restricted PPAR*β* */* *δ* deletion in heart of mice reduced myocardial fatty acid oxidation and the mRNA expression of genes involved in this process, such as PDK-4, and led to cardiomyopathy [86]. The mechanism by which activation of NF-*κ*B results in reduced expression of PPAR*β* */* *δ* target genes seems to involve reduced interaction of this PPAR subtype with its *cis*-regulatory element, since NF-*κ*B activation caused a dramatic reduction in the binding of PPAR*β* */* *δ* protein to the PPRE probe. This reduction was partially reversed by co-incubation of the cells with NF-*κ*B inhibitors, which confirms the involvement of this transcription factor in the changes observed. Therefore, the reduced binding activity of PPAR*β* */* *δ* seems to be related to the activation of NF-*κ*B in cardiac cells. However, the mechanism by which NF-*κ*B activation prevented the interaction of PPAR*β* */* *δ* with its response element still had to be established. NF-*κ*B is present in the cytoplasm as an inactive heterodimer that consists mostly of the p50 and p65 subunits. However, after activation, this heterodimer translocates to the nucleus and regulates the expression of genes involved in inflammatory and immune processes. Our results indicated that once the p65 subunit of NF-*κ*B reaches the nucleus it interacts with PPAR*β* */* *δ* *.* This association prevents PPAR*β* */* *δ* from binding to its response element, and thereby inhibits its ability to induce gene transcription, which leads to a reduction in the expression of PDK-4. In a recent study it has also been reported that PPAR*β* */* *δ* ligands and overexpression of this nuclear receptor suppressed myocardial inflammatory responses, such as the lipopolysaccharide-mediated production of TNF*α*. This had beneficial effects on animals that had undergone ischemia/reperfusion injury or cardiac hypertrophy [87].

## 7. PPAR**β**/**δ**, Inflammation, and Atherosclerosis

There is a strong relationship between circulating lipoproteins and atherosclerosis, since modified LDL-cholesterol particles are taken up by monocyte-derived macrophages, which leads to the formation of what are known as foam cells in the arterial intimal wall. Furthermore, these macrophages contribute to the inflammatory reaction by the production and secretion of numerous proinflammatory cytokines [88].

Treatment of obese rhesus monkeys, which are a model for human obesity and its associated metabolic disorders, with the PPAR*β* */* *δ* agonist GW501516 increased HDL-cholesterol (79%), and decreased triglycerides (56%), LDL-cholesterol (29%), and fasting insulin levels (48%) [22]. A decrease in proatherogenic small dense LDL was also observed in treated animals [89]. It has been suggested that the increase in HDL-cholesterol levels after PPAR*β* */* *δ* treatment is caused by enhanced cholesterol efflux, which is stimulated by higher expression of the reverse cholesterol transporter ATP-binding cassette A1 (ABCA1) in several tissues, including human and mouse macrophages and intestinal cells and fibroblasts [90, 91]. Apart from these beneficial effects of PPAR*β* */* *δ* activation on HDL levels, treatment with this compound also increased HDL particle size in primates [92]. This effect is thought to be protective against the progression of coronary artery disease in humans [93]. In addition, PPAR*β* */* *δ* activation reduces cholesterol absorption through a mechanism that may at least partly involve reduced intestinal expression of Niemann-Pick C1-like 1 (*Npc1l1*), which is the proposed target for ezetimibe, an inhibitor of cholesterol absorption [91]. Furthermore, deletion of PPAR*β* */* *δ* in mice led to enhanced LDL and triglyceride levels [94]. The administration of a PPAR*β* */* *δ* agonist to obese and diabetic *db/db* mice slightly increased HDL particles, without affecting triglyceride levels [90]. In a shorter treatment with GW501516, a reduction in plasma free fatty acids and triglyceride levels was observed in *db/db* mice, but not in mice exposed to a high-fat diet [95]. In humans, there are conflicting reports on whether PPAR*β* */* *δ* polymorphisms are associated with changes in plasma lipoproteins. Thus, while some authors found an association between a PPAR*β* */* *δ* polymorphism and plasma lipids [96], this was not confirmed by others [97]. These discrepancies could be caused by differences in sex or the influence of gene-environment interactions, since a recent study reported that the association between the PPAR*β* */* *δ*-87T>C polymorphism and plasma HDL-cholesterol might be sex-specific, with women showing a stronger association. This association was only observed in subjects consuming a low-fat diet [98]. The authors concluded that the presence of the PPAR*β* */* *δ*-87T>C polymorphism, which may result in enhanced PPAR*β* */* *δ* activity, is associated with lower risk of suffering from metabolic syndrome and that this association depends on the amount of fat consumed. In summary, the findings that are currently available on the effects of PPAR*β* */* *δ* activation on lipoprotein metabolism are so promising that PPAR*β* */* *δ* drugs are now in clinical trials for the treatment of human dyslipidemia. 

During the development of atherosclerosis, macrophages contribute to inflammation by producing and secreting numerous proinflammatory cytokines [99]. Activated macrophages express the three PPAR isotypes. Whereas the roles of the PPAR*α* and *γ* isotypes in macrophage cholesterol homeostasis are well established, the role of PPAR*β* */* *δ* remains controversial. Oliver et al. [100] showed that treatment of THP-1 human monocytes with the PPAR*β* */* *δ* ligand GW501516 enhanced the expression of the reverse cholesterol transporter ABCA1 and induced apoA1-specific cholesterol efflux. Opposite findings were reported by Vosper et al. [101]. They found that treatment with a different PPAR*β* */* *δ* ligand promoted lipid accumulation in human macrophages (THP-1) exposed to oxidized LDL by increasing the expression of the class A and B scavenger receptors (SR-A and CD-36) and the lipid storage-related genes *aP2* and *adipophilin* [101]. Finally, two studies showed that cholesterol efflux or accumulation was not affected by PPAR*β* */* *δ* depletion or by PPAR*β* */* *δ* ligands in murine macrophages [102, 103]. Collectively, these findings suggest that PPAR*β* */* *δ* does not affect cholesterol metabolism in mice. However, additional studies are needed to establish the role of this nuclear receptor in human macrophage cholesterol metabolism.

It is accepted that inflammation links dyslipidemia to atherosclerotic plaque formation [104]. Several studies have described a role for PPAR*β* */* *δ* in inflammation in atherosclerosis. For instance, atherosclerosis-prone LDL receptor-null mice transplanted with bone marrow from *P* *P* *A* *R* *β*/*δ*-deficient mice and fed a high-cholesterol diet showed smaller vascular lesions (50% reduction) than wild-type recipient controls [102], whereas no significant differences between the two groups were found for plasma cholesterol levels. However, PPAR*β* */* *δ*-null macrophages had lower expression of the inflammation markers MCP-1, interleukin 1*β* (IL-1*β*), and metalloproteinase 9 (MMP-9) [102]. In contrast, PPAR*β* */* *δ* ligands suppressed the expression of these inflammation markers in wild-type macrophages. These findings led to the suggestion that PPAR*β* */* *δ* regulates an inflammatory switch by binding or releasing the anti-inflammatory transcriptional suppressor protein B cell lymphoma-6 (BCL-6) [72, 102]. In the absence of the ligand, PPAR*β* */* *δ* sequesters BCL-6, which leads to inflammation. However, in the presence of the ligand, PPAR*β* */* *δ* releases BCL-6, which then represses inflammatory gene expression. Similarly, deletion of PPAR*β* */* *δ* also releases BCL-6, which has anti-inflammatory effects. Therefore, this mechanism may explain how both PPAR*β* */* *δ* activation and the deletion of this nuclear receptor result in a similar reduction of inflammation. Whether additional mechanisms may contribute to the anti-inflammatory effect of the PPAR*β* */* *δ* isotype remains to be studied. It should be noted that part of the anti-inflammatory effects of PPAR*γ* agonists used at high concentrations in macrophages has been attributed to the activation of PPAR*β* */* *δ* [105], since at high concentrations, PPAR*γ* ligands may activate both PPAR isotypes. Although these findings indicate that PPAR*β* */* *δ* activation may be beneficial in the treatment of atherosclerosis, *in vivo* studies are contradictory. Thus, Li et al. [103] reported no effect of the PPAR*β* */* *δ* agonist GW0742 on atherosclerotic lesion size in male LDL^−/−^ mice fed an atherogenic diet (1.25% cholesterol). In contrast, Graham et al. [106] reported that administration of GW0742 to female LDL^−/−^ mice fed an atherogenic diet (0.25% cholesterol) reduced atherosclerosis by 30%. Differences between these two studies (sex, cholesterol supplementation, or drug doses) may explain the contradictory results. Overall, these data suggest that PPAR*β* */* *δ* agonists have anti-inflammatory effects *in vivo*, but not sufficient to inhibit the development of atherosclerosis in extreme hypercholesterolemic animal models of the disease. Additional studies will be needed to determine the exact role of PPAR*β* */* *δ* in modulating the development of atherosclerosis can be determined.

## 8. Concluding Remarks

The treatment and prevention of obesity, insulin resistance, and type 2 diabetes mellitus requires lifestyle changes, including weight reduction, increased physical activity and diet. However, many patients cannot control these pathologies with lifestyle modification and there is a need for drugs to manage them. Activation of PPAR*β* */* *δ* may become a pharmacological strategy for treating these disorders. This treatment improves atherogenic dyslipidemia by reducing plasma triglyceride levels and enhancing plasma HDL-cholesterol levels. PPAR*β* */* *δ* also regulates the availability of BCL-6, an inflammatory suppressor protein that is released upon ligand binding to PPAR*β* */* *δ*, thereby behaving as an “anti-inflammatory switch” to control macrophage-elicited inflammation and atherogenesis. In skeletal muscle, PPAR*β* */* *δ* ligands may also upregulate fatty acid transport and oxidation, which reduces fatty acid-induced inflammation and insulin resistance. In adipose tissue, they prevent the activation of NF-*κ*B by reducing the production of inflammatory cytokines. In addition, PPAR*β* */* *δ* activation in the heart prevents cardiac hypertrophy and improves cardiomyopathy. 

As with any drug designed for human therapy, a great deal of research will be needed on the efficacy and safety of PPAR*β* */* *δ* activators before they reach clinical use. For instance, the ability of PPAR*β* */* *δ* activators to raise HDL-cholesterol levels in rodents has been demonstrated in primates, but the effects of these drugs on the prevention of obesity in rodents were not observed in primates. This suggests that weight reduction caused by PPAR*β* */* *δ* ligands in mice depends on their effects on thermogenesis, which is a minor mechanism of energy expenditure in humans and primates. Safety issues have also been raised regarding the connection between PPAR*β* */* *δ* ligands and carcinogenesis, particularly in animal models [107–110]. However, synthetic PPAR*γ* and *α* ligands induce carcinogenesis in rodents, but do not present these effects in humans [111, 112]. In summary, clinical studies are required to determine the efficacy and safety of PPAR*β* */* *δ* ligands.
